# Vehicle Detection in Remote Sensing Image Based on Machine Vision

**DOI:** 10.1155/2021/8683226

**Published:** 2021-08-09

**Authors:** Liming Zhou, Chang Zheng, Haoxin Yan, Xianyu Zuo, Baojun Qiao, Bing Zhou, Minghu Fan, Yang Liu

**Affiliations:** ^1^Henan Key Laboratory of Big Data Analysis and Processing, Henan University, Kaifeng, Henan, China; ^2^School of Computer and Information Engineering, Henan University, Kaifeng, Henan, China; ^3^Henan Engineering Laboratory of Spatial Information Processing, Henan University, Kaifeng, Henan, China

## Abstract

Target detection in remote sensing images is very challenging research. Followed by the recent development of deep learning, the target detection algorithm has obtained large and fast growth. However, in the application of remote sensing images, due to the small target, wide range, small texture, and complex background, the existing target detection methods cannot achieve people's hope. In this paper, a target detection algorithm named IR-PANet for remote sensing images of an automobile is proposed. In the backbone network CSPDarknet53, SPP is used to strengthen the learning content. Then, IR-PANet is used as the neck network. After the upper sampling, depthwise separable convolution is used to greatly avoid the lack of small target feature information in the convolution of the shallow network and increase the semantic information in the high-level network. Finally, Gamma correction is used to preprocess the image before image training, which effectively reduces the interference of shadow and other factors on training. The experiment proves that the method has a better effect on small targets obscured by shadows and under the color similar to the background of the picture, and the accuracy is significantly improved based on the original algorithm.

## 1. Introduction

Remote sensing target detection is to mark the object of interest in remote sensing images and then forecast the type and location of this targets. In the traditional detection dataset, the target is concentrated, while the aviation dataset is not, and the object strength in the aviation image usually appears in arbitrary orientation, which depends on the perspective of the Earth vision platform [1].

Object detection is the process of detecting instances of semantic objects of a certain class (such as humans, airplanes, or birds) in digital images and video [2]. Small target detection has always been a hot and difficult area in target detection tasks. The study of remote sensing imagery has vital applications in military, disaster control, environmental management, and transport planning [3–6]. And vehicles in remote sensing images as a special category, whether civilian, military, or transportation, have an important meaning and at the same time are more challenging.

Firstly, the small target in the target detection task is usually a target that is less than 30 pixels in the image whereas vehicle targets in remotely sensed images are usually below 20 pixels or even 10 pixels. Secondly, the class of vehicle in remotely sensed images is often subject to weather and environmental images such as atmospheric occlusion, shadow occlusion, and building occlusion and other factors, for example, different overhead views, different sizes of vehicle targets in the same image, similar colors between vehicles and their surroundings, and so on. It resulting in poor detection accuracy of car targets.

For the past few years, followed by the rapid science development of deep learning, great progress in target detection methods has been made. Among them, the popular target detection methods based on the convolutional neural network [7–12] can be divided into one stage [7–9, 13–18] and two stages [8, 19–24]. One stage, such as YOLO [9], SSD [7], DSSD [13], YOLOv2 [17], YOLOv3 [18], and Retina Net [16] and so on. The one-stage algorithm has a very fast speed in the detection process, and it has a widespread application in each kind of scenes which needs high efficiency. In the traditional target dataset, the one-stage algorithm in the process of detection always has a fast speed and high accuracy. However, when applied to optical remote sensing datasets, its accuracy will be greatly reduced. The reasons for this phenomenon are as follows: (1) optical remote sensing image is a bird's-eye view; thus, its height and shooting angles are uncertain, and (2) there are many small targets of less than 30 pixels in aerial images, but the one-stage method, such as SSD, does not perform well for small targets.

The two-stage algorithms, such as RCNN [20], SPP net [24], fast RCNN [21], faster RCNN [19], and Mask R-CNN [23], divide the detection task into two subtasks: recognition and localization. Compared with one-stage algorithms, the accuracy of the two-stage algorithm is higher, but their training time is often longer, and the detection speed is relatively slow.

Since the YOLO series algorithm was put forward, its speed has been widely affirmed, but the accuracy is relatively poor. The latest YOLOv4 algorithm has greatly improved in accuracy and has been widely used in many fields. The backbone network of the YOLOv4 algorithm improves the darknet53 network proposed in the YOLOv3 algorithm by adding CSP[25] module, and SPP enhanced network [24] is added after the backbone network. PANet [26] is used for the neck network of the algorithm, and YOLO head is still used as the detector. In the PANet structure [26], the algorithm divides the feature map into three scales according to the scale size, namely,19 × 19, 38 × 38, and 76 × 76 for detection. Upsampling is carried out at the two adjacent scales, and then downsampling is performed after several convolutions. Although the YOLOv4 algorithm has achieved good results so far, there is still a lot of room for improvement in its detection effect in the targets of remote sensing images, especially in the car class.

**Figure 1:**
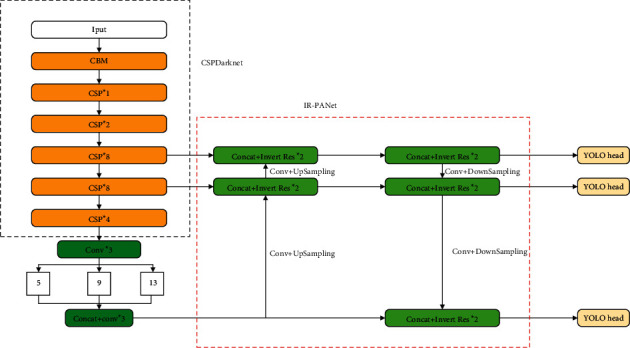
The network structure of IR-PANet with CSPDarknet.

**Figure 2:**
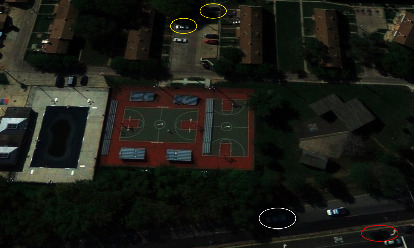
The car category in the remote sensing dataset. Where targets marked by yellow circles are vehicle targets obscured by trees, target marked by the white circle is vehicle target in shadow, and target marked by the red circle is vehicle target whose color is similar to that of the surrounding environment.

In this paper, we propose the IR-PANet algorithm for targets in shadow occlusion and targets with a similar color to their surroundings in remote sensing images. The detector replaces convolution with inverted residual [27] based on PANet, which can increase the depth of the model, enrich the semantic information of the algorithm and increase its detection accuracy. In the training, CSPDarknet53 is used as the backbone network. To test the capability of the network, we use the HRSSD [28] dataset to pretrain our model. Considering that vehicles in remote sensing images are easily obscured by shadows, we apply GAMMA correction to the image for brightening the image and removing noise. The main contributions of this study are as follows:

1. Given the particularity of remote sensing images, an IR-PANet is proposed and applied to the YOLOv4 method. The recognition ability of the model for small targets and occluded targets is increased. In this method, we replace the original convolution with inverted residual after upsampling, and the improved algorithm is shown in Figure 1. In our method, the network becomes deeper without much increase in computation, which greatly deepens the robustness of the network and increases the network semantic information.
2. The original image is preprocessed. Before training, Gamma correction is used to preprocess the image, which reduces the noise in the original image, brightens the shadow part of the image, and improves the recognition rate of the shadow-covered target. The processed image is shown in Figure 2.
3. YOLOv4 algorithm has a low recognition rate for targets with complex backgrounds. In this paper, we compare two classes of the HRSSD dataset, vehicle class and ship class. Among them, the vehicle data and background are complex and have shadow, building occlusion, incomplete semantic information, dense target, and so on. However, the background of ship data is simple and the target is single. Proved after the experiment, the accuracy of the original algorithm in the vehicle dataset is not high, while the accuracy of the IR-PANet algorithm in the vehicle dataset has been significantly improved after training.

The rest of this paper is organized as follows: Section 2 introduces related research on vehicle detection in common images and remote sensing images.  Section 3 describes our proposed model of remote sensing image target detection.  Section 4 describes the experimental design and experimental details.  Section 5 describes the experimental results and analysis. Finally, we conclude this study in section 6.

## 2. Related Work

While existing algorithms have achieved good results in detection tasks, much work has been done to improve them for the characteristics of vehicle targets. For example, Jun et al. [29] proposed a vehicle detection model YOLOv2_vehicle based on the YOLOv2 algorithm and obtained an average accuracy of 94.78% on the Beijing Institute of Technology (BIT) Vehicle validation dataset. Hu et al. [30] proposed a scale-insensitive convolutional neural network (SINet) for fast detection of vehicles with large scale variance. Nguyen [31] proposed an improved fast R-CNN based framework for fast vehicle detection. Xu et al. [32] proposed a Side Fusion FPN algorithm for application in the Resnet-86 backbone network to detect vehicles and pedestrians on the road.

Deep learning continues to have a wide range of applications in remote sensing image target detection tasks. For instance, Deng et al. [33] proposed a unified and effective method for simultaneous detection of multiple classes of targets in large scale change remote sensing images by using multiscale object proposal network (MS-OPN) and an accurate object detection network (AODN) for target classification and detection and achieved higher accuracy in datasets such as NWPU VHR-10. Zhang et al. [34] proposed a dual multiscale feature pyramid network (DM-FPN) by combining strong semantics, low-resolution features, and weak semantics using the inherent multiscale pyramidal features of remote sensing images. Eten et al. [35] improved a network for raw resolution detection of remote sensing data based on YOLOv2 and concluded that objects as small as 5 pixels in size can still be located at high resolution. Ming et al. [36] introduced the CFC-NET detection network, which optimizes the single-stage detector in terms of feature representation, anchor refinement, and training sample selection.

For vehicle-based targets in remote sensing images, Tang et al. [6] used an R-CNN network-based approach for real-time monitoring of remote sensing images of vehicle targets. Gao et al. [37] proposed a novel detection model, DE-CycleGAN, to enhance weak targets and achieve accurate remote sensing image detection. Zhang et al. [38] proposed a three-step local proposal method (LRP) for the detection of live vehicles in satellite video. Shi et al. [39] proposed a single-stage and anchorless detection method to detect oriented vehicles in high-resolution aerial images by linking coarse and fine feature maps output from different stages of the residual network through a feature pyramid fusion strategy.

## 3. Materials and Methods

In this section, we first introduce the used backbone network CSPDarknet and, then, the structure of the inverted residual block and its advantages. Then, the next section introduces our proposed IR-PANet network structure. Finally, we detail the image preprocessing method, GAMMA correction.

### 3.1. Backbone Network

The backbone network used in this paper is CSPDarknet [40]. YOLO-based target detection algorithms say that detection problems are defined as single regression problems; that is, a single neural network can achieve probability prediction of multiple enclosing boxes and classes for an entire image through a positive operation. Based on YOLOv3, CSPDarknet53 introduces the CSP module based on Darknet53. Before each downsampled, it will carry out semantic fusion with the top-level information to increase network depth and enrich the network. Each residual module consists of convolution layers of 1×1 and 3×3 and a quick connection. Convolution structure is composed of convolution layer, batch normalization, and mish [41] activation function.

**Figure 3:**
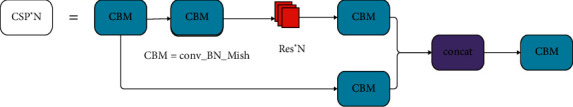
The network structure of the CSP module.

CSPDarknet53 attributes the problem to repeated gradient information in network optimization and focuses on the variability of the gradient by integrating feature mapping from the beginning and end of the network control phase [25]. The structure of the CSP module is shown in Figure 3.

### 3.2. The Module of Invert Residual

In the remote sensing image, the content is complex. All these lead to the loss of semantic information in the image during the convolution process. Objects like vehicles are easily affected by clouds, shadows, buildings, and other factors. The principle of invert residual is to replace convolution with depthwise separable convolution, deeply separate information, fuse channels through multilayer 1*∗*1 convolution, and finally restore to the target size, in which each block contains an input, the middle is several bottlenecks, and then it is the expansion. However, the bottleneck contains all the necessary information, and the extension layer is only used as the implementation details of the neighborhood tensor nonlinear transformation. We directly use the shortcut between the bottlenecks [27].

In the invert residual module, we still use LeakyRelU as our activation function, and the expression is as follows:

(1) $$\begin{array}{c} Leaky\;Re\;LU\left(x\right)=\left\{\begin{array}{cc} x, & x\geq 0,\\ ax, & x\leq 0, \end{array}\right.\in R \end{array}$$

where *x* is the input information. In invert residual, the total number of multilayer additions required for blocks is (*m*/*s*) × (*n*/*s*) × (*tc*) with the input size of *m* × *n*, *t* is the expansion factor, and *c* is the core size. As shown in Table 1, compared with a convolution, the amount of computation is greatly reduced. The input and output parameters for the calculation of standard convolution and inverse residual are shown in Table 1.

**Figure 4:**
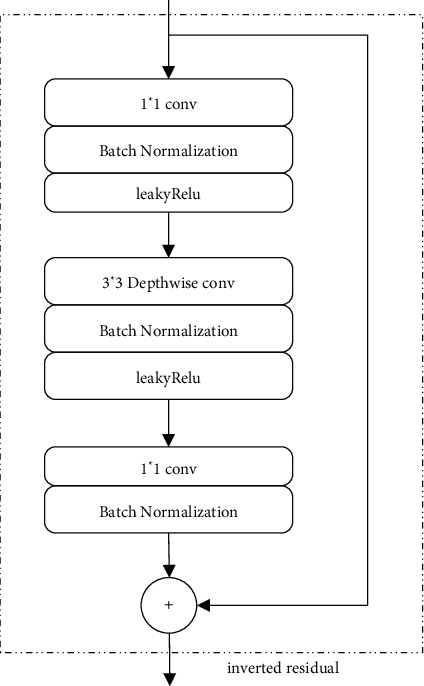
The network structure of the inverted residual module.

The interface diagram of the inverted residual module is shown in Figure 4.

### 3.3. The Network Structure of IR-PANet

The high-level network has a strong sense of the whole image, while other neurons are more likely to be activated by local texture and pattern [26], which indicates that it is necessary to increase the top-down path to spread semantic features to enhance the robustness of the network.

PANet network first follows the definition of FPN, which has upsampling from bottom to top, enriching the feature information of the upper network detection layer, while CSPDarknet53 detection layer generates three prediction layers, which are 19×19, 38×38, and 76×76, respectively. The semantic information of the upper network is convoluted and then downsampled to the deep network, which can also enrich the semantic information of the network. This greatly improves the FPN network's ability to detect small targets. We replace the cubic convolution (1 × 1and 3 × 3 as a group) in PANet with twice inverse residual, and the output of the upper network is downsampled. Remote sensing images contain many objects with little textural information and low counterbalance. The original model is difficult to recognize complex features. The IR-PANet increases the depth of the model and reduces its calculation time, which has a positive effect on the learning process of the network. The IR-PANet network structure is shown in Figure 2.

As can be seen from Figure 2, IR-PANet performs target detection by merging feature maps of different sizes. The network upsamples each layer of the feature pyramid from top to bottom and then performs two invert residual horizontally, followed by downsampling and semantic fusion layer by layer. Besides, the output of the network contains more information about the target's location in the deep layer and the network outputs contain less information on the location of the target in the complex network. In our algorithm, we use 19×19, 38×38, and 76×76 feature maps to construct feature pyramids for large, medium, and small target detection. Prediction at multiple scales makes the algorithm have greater sensitivity to small targets and the targets in a complex environment and significantly improves its detection ability.

### 3.4. Gamma Correction

**Figure 5:**
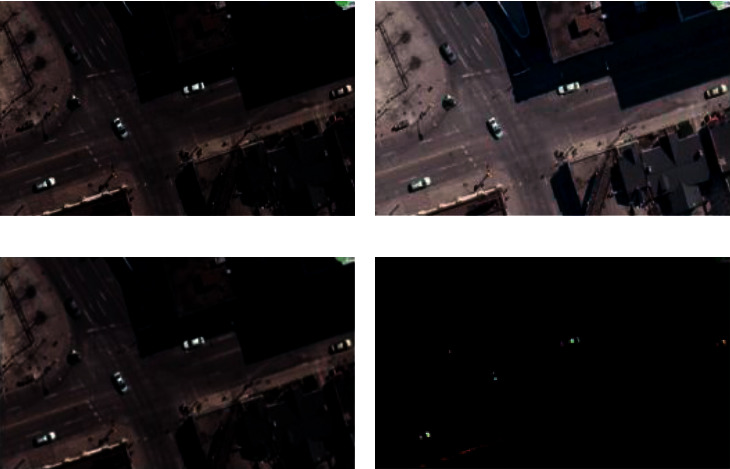
Result of the effect of gamma correction: (a) original; (b) *γ* < 1; (c) *γ* = 1; and (d) *γ* > 1.

Before we send the image into the deep learning algorithm, we first preprocess the image. As shown in Figure 5, remote sensing images are easy to receive weather, angle, building occlusion, shadow occlusion, and other factors, which affect the accuracy of our algorithm. By gamma correction, adjusting the contrast of the image, and enhancing local details, we can reduce the shadow and other factors of the image. According to [42], the formula based on Gamma correction is

(2) $$\begin{array}{c} F\left(I\right)=I^{\gamma }, \end{array}$$

where *γ* is an important adaptive parameter designed according to the principle of image chromaticity and uniform illumination intensity. As can be seen from Figure 4, when *γ* is smaller than 1, the effect is that the image almost dims, and when equaling 1, the image is not enhanced. Again, when *γ* is higher than 1, the effect of the image shows artificial facts.

## 4. Experiments Design

### 4.1. Datasets and the Evaluation Metric

#### 4.1.1. The Description of Dataset

We use the HRSSD dataset of Xi'an optical and Precision Machinery Research Institute of Chinese Academy of Sciences to verify our proposed method and select automobiles category in the dataset to evaluate. In addition, we still tested the ship class in this dataset to check the robustness of the algorithm. There are 1188 train sets, 1186 validation sets, and 2382 test sets in the automobile category and 950 train sets, 948 validation sets, and 1988 test sets in the ship category. If you want to find out more information about the dataset, please visit https://github.com/CrazyStoneonRoad/TGRS-HRRSD-Dataset.

#### 4.1.2. Evaluation Index

In this experiment, loss curve value and average precision (AP) were used for evaluating our method. The change of curve loss can reflect the error. The change in the loss curve may reflect the error between the predicted and actual results. Therefore, the faster the decrease in the loss curve and the smaller the value of the loss, the better the result of the training model. AP refers to the proportion of correct box selection in the prediction box. The higher the proportion is, the more correct box selection targets are, which indicates that the following result of the training model is better.

The loss function we use is DIoU [43]. Compared with IOU [44], DIoU directly minimizes the distance between two middle points, and the prediction box is closer to the target box. DIoU can be defined as follows:

(3) $$\begin{array}{c} {ℜ}_{DIou}=1-\text{IoU}+\frac{\rho^{2}\left(a,a^{gt}\right)}{b^{2}}, \end{array}$$

where *a* and *a*^*gt*^ are the center of the forecasting box and the target box, *ρ*() is the Euclidean distance, and *b* is the diagonal length of the shortest closed box that covers both boxes.

In multiclass target detection, mean accuracy (map) is being widely used for the evaluation index. This mAP is equal to the average of the AP values of all the categories. Its expression is as follows:

(4) $$\begin{array}{c} {\text{precision}}_{j}=\frac{{\text{TP}}_{j}}{{\text{TP}}_{j}+{\text{FP}}_{j}},\\ {\text{recall}}_{j}=\frac{{\text{TP}}_{j}}{{\text{TP}}_{j}+{\text{FN}}_{j}},\\ \text{AP}=\int_{0}^{1}{\text{precision}}_{j}\left(r_{j}\right)d\left(r_{j}\right),\\ m\text{AP}=\frac{1}{n}\overset{n}{\underset{j=1}{\sum }}AP_{j}. \end{array}$$

If the IOU of the detected box is greater than 0.5 with that of the box labeled in the actual dataset, the detected box is considered as the true position. For class *J*, TP_*j*_, FP_*j*_, and FN_*j*_ are the figures for true positions, false positions, and false negatives respectively. *n* indicates the number of HRSSD dataset classes, and *k* = 2. *r*_*j*_ is the class of recall. However, the accuracy and recall are contradictory. As the number of recalls increases, the accuracy of recall also decreases. Therefore, combining the accuracy with recall, we use mAP to evaluate our algorithm.

### 4.2. Experimental Details

In this paper, the algorithm language is C language, the operating system is Ubuntu 16.04, GPU is Quadro P4000 8GB×2, and the hardware platform is Intel (R) Xeon (R) silver 4114, CPU @ 2.2 GHz. In the process of training, the momentum is set to 0.949, and the model is optimized by asynchronous small-batch stochastic gradient descent. The initial learning rate is set to 0.0013. When the figure for training iterations is 4800 and 5400, the learning rate is adjusted to 0.00013 and 0.000013, respectively. We have iterated 6000 times of this algorithm.

In this section of the experiment, the input size of the image changes randomly in the experimental training with a multiple of 32 (the range of change is 320×320 to 608×608 pixels). The batch size is 64. Meanwhile, the training data can be increased by adjusting saturation, exposure, and tone.

## 5. Experimental Result

**Figure 6:**
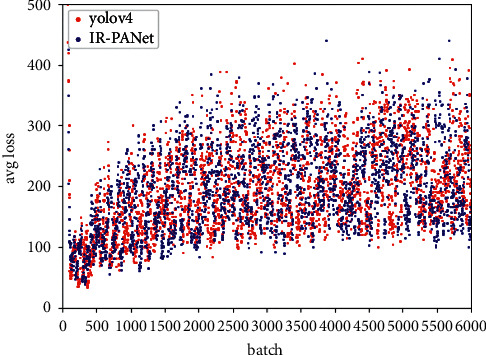
Loss comparison chart of YOLOv4 and IR-PANet.

Figure 6 is the convergence curve of the loss value of our network framework when training the ship dataset. The horizontal axis represents the figure for training iterations, the maximum iteration is 6000, and the longitudinal axis represents the loss and accuracy. From Figure 6, it is to recognize that when the number of iterations is 500, it tends to be stable. Then, we can see that the loss fluctuation interval of IR-PANet is always lower than that of YOLOv4, and its scatter is denser. Then, as can be seen in Table 2, the number of TP detected by IR-PANet is much higher than that of YOLOv4 and FN is also much lower than that of YOLOv4 while the value of precision is lower due to higher FP.

**Figure 7:**
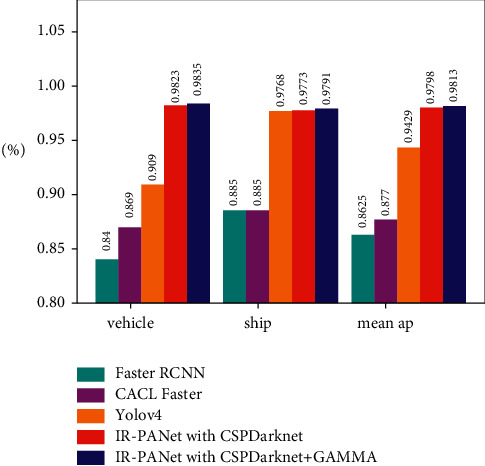
Bar chart comparison of different methods of AP and map in automobile and ship datasets.

There are 2382 test targets in the automobile dataset and 1988 test targets in the ship dataset. Among them, in the automobile dataset, the target is generally small, the distribution range is dense, the target features are occluded, and the environment is complex, resulting in the relatively high loss value of this method, but we can also see that the value of loss tends to be stable around 1000 generations. For different networks, the test results of the two classes in the HRRSD dataset are shown in Table 3 and Figure 7.

**Figure 8:**
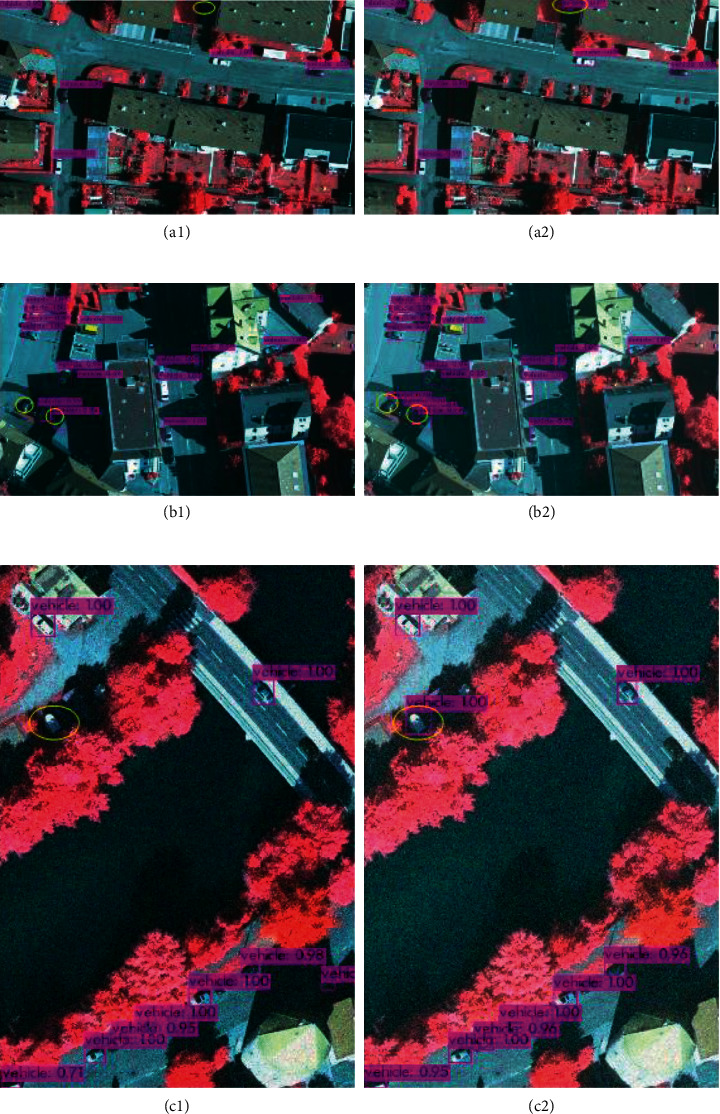
Detection results in the shadow-obscured background, where (a, c, e) are the detection results of the YOLOv4 algorithm and (b, d, f) are the detection results of IR-PANet.

In this paper, after 1188 remote sensing images are tested, it is found that the accuracy of vehicles in remote sensing images is very high, and it also has a good recognition effect in the areas with dense vehicles and shaded areas. The detection effect and accuracy are shown in Figure 8, and the contrast area is marked with a yellow box. From Figures 8(a) and 8(b), it can be found that IR-PANet detects more dense and occluded vehicle targets than IR-PANet. In Figure 8(c), the target location of missed detection is dense and shadowed with the accuracy of 78% and 84% in Figure 8(d). In IR-PANet, the accuracy of the two targets detected by YOLOv4 in these target vehicles is also increased from 100% and 99% to 99% and 94%, respectively. This indicates that IR-PANet is higher than YOLOv4 in this complex environment in detection accuracy.

**Figure 9:**
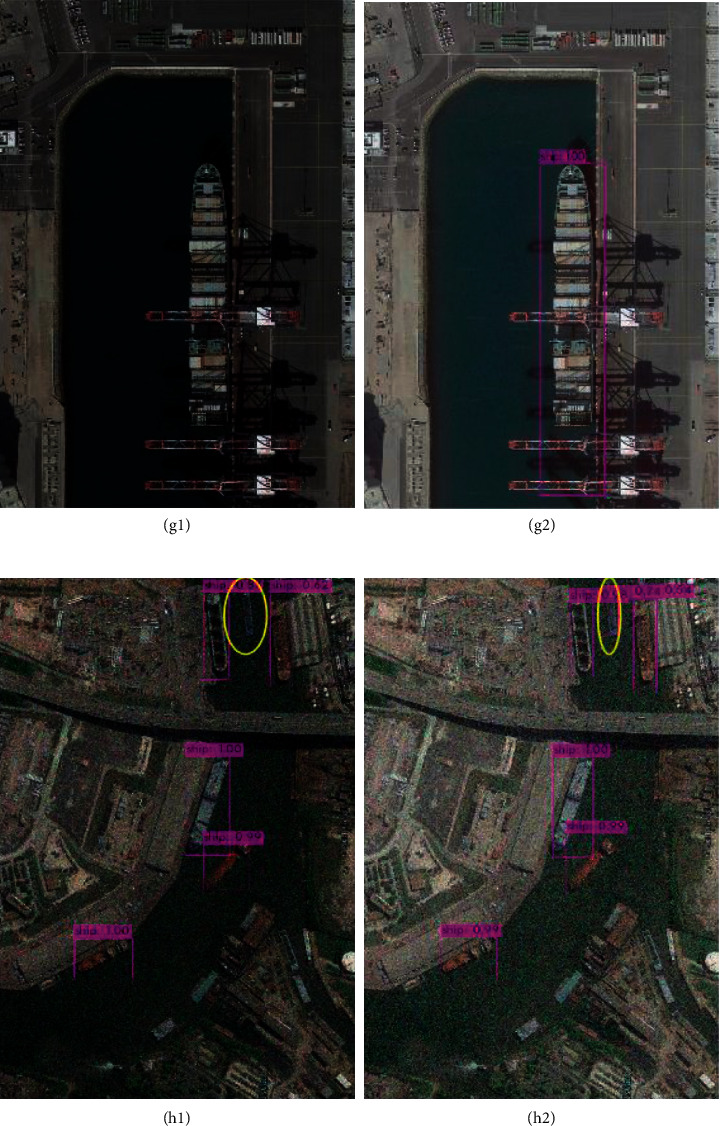
Comparison results of the YOLOv4 algorithm with the proposed algorithm in the ship class.

In the ship dataset, as a result of the large pixel size and obvious features of the target, the map of IR-PANet is similar to YOLOv4. From Figure 9(a), the left ship is affected by shadow and missed in the algorithm. In Figure 9(b), the image after Gamma correction preprocessing is completely detected. This clearly shows that after Gamma correction, the algorithm is easier to learn useful feature information.

In the following, we will show the comparison result graphs of YOLOv4 and the proposed algorithm in terms of targets in a shadow-obscured environment and dark target miss detection.

Target shadow occlusion is a difficult problem for target detection in remote sensing images, and as can be seen in Figure 8, the proposed algorithm works better in this scene. It is also clear from Figures 8(e) and 8(f) that the detection of targets in shadow is more accurate.

**Figure 10:**
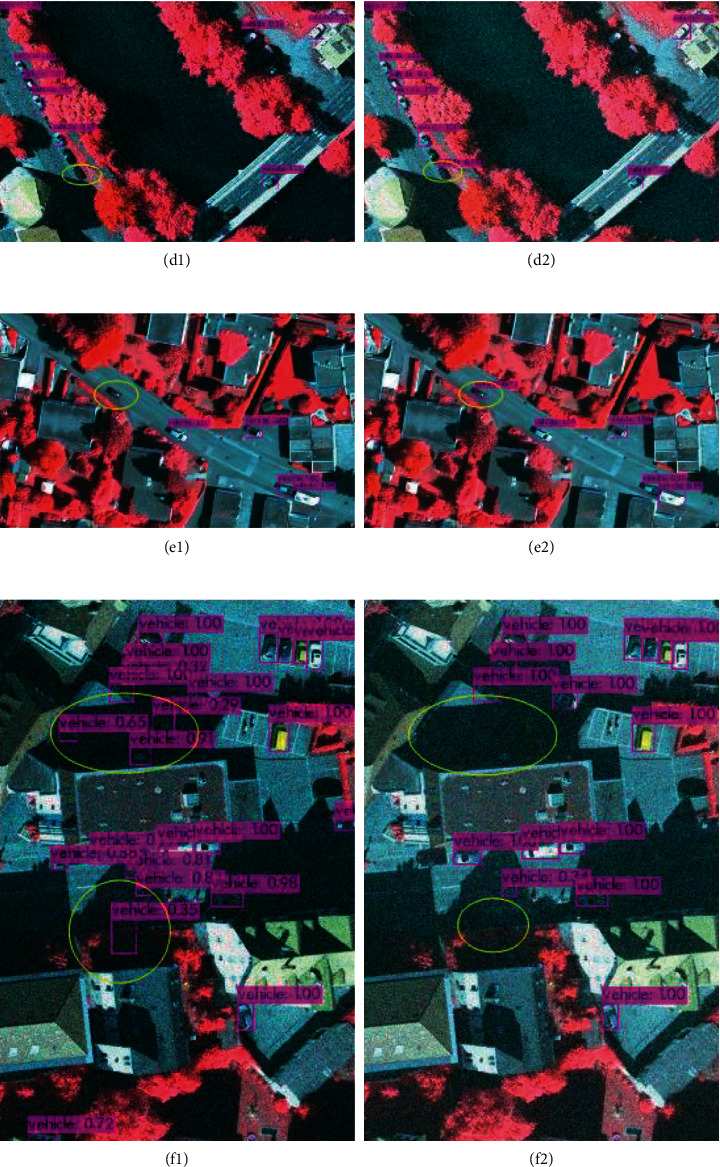
The results of dark target detection are compared, where (a, c, e) are the detection results of YOLOv4 and (b, d, f) are the detection results of IR-PANet, where the comparison results of (e and f) can see that YOLOv4 is easier to identify the shadow block as a target, while IR-PANet is more accurate.

The dark target features in remote sensing images are close to the shadows as well as the picture background, which is also easy to miss and error. It can be seen from Figure 10 that IR-PANet has slightly better accuracy than YOLOv4 in identifying dark targets, while Figures 10(e) and 10(f) demonstrate that the proposed algorithm is more accurate in detecting shadows clearly.

In the ship class, the target is less affected by the environment; as can be seen in Table 3, the map of IR-PANet and YOLOv4 algorithm is close. And from Figure 9, the ship target in g1 receives shadow occlusion, while the image is clearer after image processing, and our algorithm is better recognized. In Figures 9(c) and 9(d), the detection effect of the target color is similar to the background color which can also be better shown. This fully illustrates that IR-PANet has strong robustness.

## 6. Conclusions

We propose a new CNN structure, IR-PANet, to identify vehicle targets in optical remote sensing imagery. The network is applied in the CSPDarknet backbone. In the detection layer, we improve the network by making it deeper and larger. Before training, the images are preprocessed with GAMMA correction and the appropriate number of anchor boxes is reset to provide better target detection performance in difficult environments such as shadows. In the experiments, IR-PANet which is running under Quadro P4000 obtained the achievement of *ap*=98.35% in the vehicle class, improving the accuracy by 7.45% with a loss of 3.8 fps detection speed, outperforming other neural networks. However, the experimental results indicated that the number of FP (the number of actual negative classes predicted as positive classes) was higher for IR-PANet. This is an area for improvement in our future research. In addition, experiments have shown that the performance of IR-PANet is demonstrated in the ship class. By evaluating the optimization of the proposed network models for small objectives (cars and ships) in the context of two different complexes, this study is relevant to the research of aerial remote sensing image detection technology.

## Figures and Tables

**Table 1:** Comparison of standard convolution and inverse residual.

Input	Operator	Output
*m* × *n* × *c*	1*∗*1 convolution	*m* × *n* × (*tc*)
*m* × *n* × (*tc*)	3*∗*3 invert residual	(*m*/*s*) × (*n*/*s*) × (*tc*)

**Table 2:** Comparison of TP (the number of actual positive classes predicted as positive classes), FP (the number of actual negative classes predicted as positive classes), FN (the number of actual positive classes predicted as negative classes), *F*1-score, recall, and precision of YOLOv4 and IR-PANet in vehicle classes

	YOLOv4	IR-PANet
TP	2029	**2284**
FP	123	404
FN	294	**39**
*F*1-score	0.91	0.91
Recall	0.87	0.98
Precision	0.94	0.85

**Table 3:** Results of Faster RCNN [45], CACL Faster RCNN [45], YOLOv4, IR-PANet, and IR-PANet with GAMMA in the dataset.

	Vehicle	Ship	mAP	FPS
Faster RCNN	0.84	0.885	0.8625	—
CACL Faster RCNN	0.869	0.885	0.877	—
YOLOv4	0.9090	0.9768	0.9429	19
IR-PANet	0.9823	0.9773	0.9798	15.2
IR-PANet with GAMMA	**0.9835**	**0.9791**	**0.9813**	15.2

## Data Availability

The data are available at https://github.com/CrazyStoneonRoad/TGRS-HRRSD-Dataset.
